# Policy: Framing a Chemical Future

**Published:** 2005-07

**Authors:** Richard Dahlz

Spurred by recent developments abroad to design new approaches to chemical management, the University of Massachusetts Low-ell Center for Sustainable Production sponsored a two-day conference in April 2005 to stimulate similar productive changes in the United States. The event attracted a mix of some 170 environmentalists, government officials, academics, product representatives, and chemical industry representatives.

The conference aimed to initiate the kind of multi-stakeholder dialogue that the European Commission created in the late 1990s. The European effort has resulted in a draft regulation called REACH (Registration, Evaluation and Authorisation of Chemicals), which calls for manufacturers and importers to identify and report the properties of the substances they use and sell. Other international actions, such as the 2002 World Summit on Sustainable Development, which set a goal of achieving sound global management of chemicals by 2020, have also heightened the need for better U.S. chemical policies. Discussions begun at this workshop may eventually lead to public policy supporting a safer and more competitive U.S. chemical industry.

Speakers opened the meeting with talks on the current thinking of chemical policy as well as specific policies and industrial protocols. Then participants broke out into workshops on various subjects. One group focused on the promotion of innovative industry initiatives, “green chemistry,” and alternative materials. Others dealt with improving information flows in supply chains and beyond, integration of U.S. and global chemical initiatives, and incorporation of improved chemicals management into business processes.

One theme repeated throughout the conference was the need for improved communication about what’s in the products that people buy, and greater transparency of the people who make the products. “The thing that I really noticed about [participant feedback] was the importance that people placed on information—the flow of information, the access to information, who’s responsible, where it’s stored,” said center director Kenneth Geiser.

A second repeating theme was support for green chemistry, chemical processes that reduce or eliminate the use and generation of hazardous substances in economically viable ways. Paul Anastas, director of the Green Chemistry Institute in Washington, DC, said he noticed a recognition that such scientific innovations “can be both economically profitable and environmentally preferable.”

Geiser said there also was nearly universal agreement by participants that the current U.S. chemicals policy, largely embodied in the Toxic Substances Control Act of 1976, is outdated. “Almost everyone I talked to felt that the current chemicals policy system needs overhauling,” he said. “It’s interesting that the business folks felt the same way; it’s not working for them, either.”

The Lowell Center will compile a report on the conference in the next few months. Geiser believes the conference’s goal has been met. “I think we created an enthusiasm for moving forward,” he said. “That was pretty much what we wanted.”

